# Primate vocal production and the riddle of language evolution

**DOI:** 10.3758/s13423-016-1076-8

**Published:** 2016-07-01

**Authors:** Julia Fischer

**Affiliations:** Cognitive Ethology Laboratory, German Primate Center, Kellnerweg 4, 37077 Göttingen, Germany

**Keywords:** Alarm calls, Primate communication, Referential signalling, Speech evolution

## Abstract

Trying to uncover the roots of human speech and language has been the premier motivation to study the signalling behaviour of nonhuman primates for several decades. Focussing on the question of whether we find evidence for linguistic reference in the production of nonhuman primate vocalizations, I will first discuss how the criteria used to diagnose referential signalling have changed over time, and will then turn to the paradigmatic case of semantic communication in animals, the alarm calls of vervet monkeys, *Chlorocebus pygerythrus*. A recent in-depth analysis of the original material revealed that, while the alarm calls could be well distinguished, calls of similar structure were also used in within- and between-group aggression. This finding is difficult to reconcile with the idea that calls denote objects in the environment. Furthermore, nonhuman primates show only minimal signs of vocal production learning, one key prerequisite for conventionalized and symbolic communication. In addition, the structure of calls in different populations or closely related species is highly conserved. In conclusion, any continuity between nonhuman primate and human communication appears to be found at the level of the processing of signals. Why and how the ancestors of our own species one day began to talk to each other continues to be an enigma. Future research should focus on changes in the neural structure supporting volitional control over vocalizations, the gene networks associated with vocal production, and the developmental processes involved in the integration of production and perception of vocalizations.

Are animal vocalizations just expressions of emotions (Darwin 1872) or do they perhaps have a symbolic component, as the eminent ethologist Peter Marler suggested (Marler, 1977)? Can we find rudimentary forms of what we consider to be prerequisites for—or instances of—linguistic abilities in animal communication (Fitch & Zuberbühler, 2013; Fitch, 2010)? Over the last decades, we have learnt a great deal about nonhuman primate communication. Yet, I suggest that we are still pretty much in the dark when it comes to understanding the evolution of language as a representational system (Hagoort & Poeppel, 2013), as well as the evolution of the flexibility in vocal production that is necessary to develop a conventionalized verbal communication system.

To understand how the field of primate communication developed, let me turn back the clock to the year 1980, when Robert Seyfarth, Dorothy Cheney, and Peter Marler published their seminal paper on the alarm-calling behaviour of vervet monkeys, *Chlorocebus pygerythrus* (previously *Cercopithecus aethiops*) (Seyfarth, Cheney, & Marler, 1980a). Building on Tom Struhsaker’s (1967) thorough account of the vocal behaviour of this species, which indicated that vervet monkeys give different alarm calls in response to different predator classes, Seyfarth et al.’s playback experiments revealed that listeners typically selected appropriate predator avoidance behaviours when hearing specific call types, even in the absence of the predator itself (Seyfarth et al., 1980a). The finding that subjects would be able to interpret the calls correctly, even in the absence of any contextual cues, revolutionized the field. “The monkeys responded as though each type of alarm call designated different external objects of events”, Seyfarth and colleagues noted (Seyfarth et al., 1980a, p. 803), and concluded that the vervet alarm calls could be viewed as rudimentary semantic signals, although they also cautioned that “There are limits to how far a semantic analysis can be carried out when it is based solely on the responses that those signals evoke” (Seyfarth, Cheney, & Marler, 1980b, p. 1091). Nevertheless, the idea that monkey vocalizations refer to events in the environment stood in stark contrast to earlier conceptions of primate vocalizations as manifestations of arousal, and put the question of animal semantics at the intersection of evolutionary theory, linguistics and semiotics.

About a decade later, Marler and colleagues pointed out that the similarity to human linguistic reference was restricted to the receiver side (Marler, Evans, & Hauser, 1992), and that the cognitive underpinnings of the signalling behaviour remained largely unclear. Because listeners were apparently able to retrieve information from the calls about the environment, such as the appearance of a martial eagle in the sky, Macedonia & Evans (1993) coined the term ‘functional reference’—the calls *functioned as if* the signallers referred to these events. The diagnostic criteria for functional reference were acoustic distinctiveness and production specificity at the side of the sender, and differential responses at the side of the receiver. Based on these criteria, numerous studies identified ‘functional reference’ in animal calls in a variety of taxa, including primates, rodents and birds (see Townsend & Manser, 2013; Wheeler & Fischer, 2012, for reviews). Although the mental processes underpinning the production of (more or less) context-specific calls remained unclear, the idea prevailed that these alarm signals are in some way informative for understanding language evolution. Indeed, functional reference continues to be singled out as providing insights into the evolution of language to the present day (Watson et al., 2015b), and, to a number of scholars, the concept is worth defending (Scarantino & Clay, 2015; Scarantino, 2014; Townsend & Manser, 2013).

Remarkably, although the investigation of the vervet alarm calls is the classic study in this field, it was not clear how context-specific these calls really were. Previous analyses had focused on only a subset of calls (Owren & Bernacki, 1988). Therefore, Tabitha Price, then a PhD student in our group, initiated a comprehensive analysis of vervet calls (Price et al., 2015). This analysis made use of the material collected by Struhsaker, Seyfarth and Cheney, and included not only alarm calls given in response to different predator classes but also calls given during inter-group and intra-group aggression. This was based on the observation that vervet monkeys produce so-called ‘chutters’, not only in response to snakes but also during inter-group aggression (Cheney, 1984; Struhsaker, 1967). Further, we added the ‘bark’-like calls that Tabitha Price had recorded from South African male vervets; these calls had been given in response to real and visual model leopards, and during within- and between-group encounters. Because of substantial differences in the structure of male and female calls, we ran separate analyses for each sex.

To assess the context specificity of female calls, we first considered only those calls that were given in predator contexts. Based on a discriminant function analysis, 98.7% of female calls were correctly assigned to the respective context (Fig. 1a), indicating that the alarm calls per se could be well distinguished based on their acoustic features. When we added calls recorded during aggressive interactions, however, the assignment to the contexts dropped to 71.4 % correct classification. Misclassification occurred between calls given during within- and between-group aggression, and eagle and snake contexts. Overall, we found that the female calls given in response to leopards were clearly distinct, but found continuous variation between ‘chutters’ and ‘rraups’ (Fig. 1b).

**Fig. 1:**
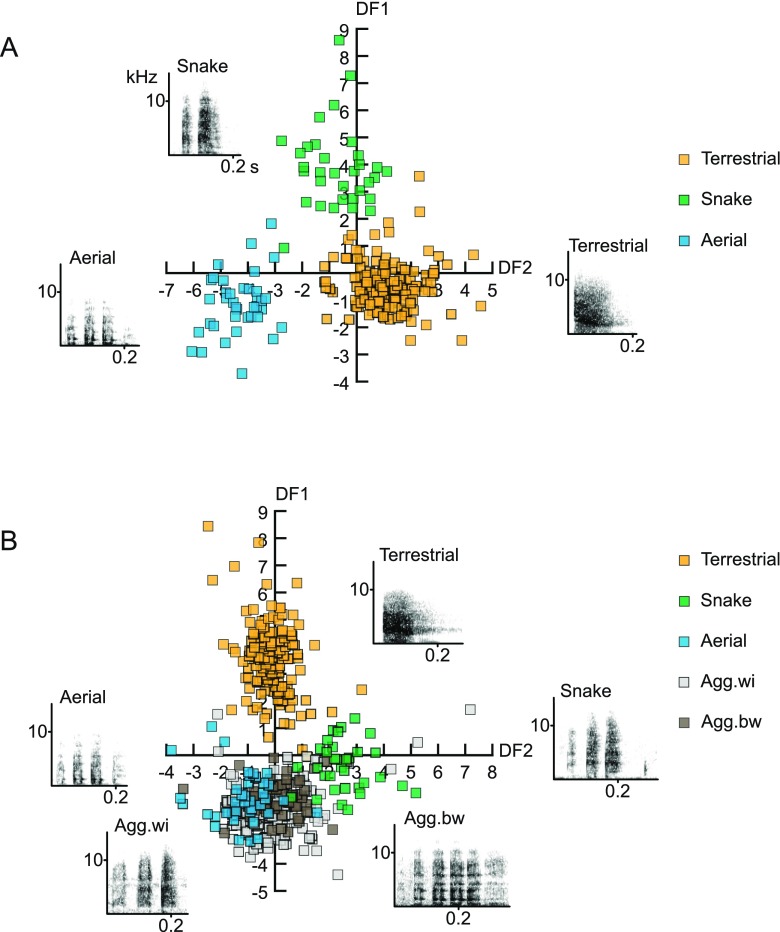
Structure and discriminability of female vervet monkey, *Chlorocebus pygerythrus*, vocalizations given in alarm and aggressive contexts. (**a**) Scatter plot of the discriminant scores with corresponding spectrograms of female alarm calls given in response to leopards, eagles, and snakes. (**b**) Scatter plot of the discriminant scores with corresponding spectrograms of female alarm calls given in response to leopards, eagles, and snakes, as well as during within- and between-group aggression. *DF* discriminant function. Modified from Price et al., 2015

For the East African male alarm calls, the vocalizations given in response to different predators could also be well distinguished, with 93.2 % correct classification (Fig. 2a). Because no calls from aggression contexts were available, we compared male barks recorded from South African male vervet monkeys that were given in response to leopards and during intergroup aggression (Fig. 2b). Calls in these two contexts were relatively similar to each other, resulting in an average correct classification of 74.9 %. In sum, we corroborated that the alarm calls of East African vervet monkeys are acoustically well distinguishable. Yet, when additional contexts were considered, notable misclassification occurred for the ‘chutter’ and ‘rraup’ calls.

**Fig. 2:**
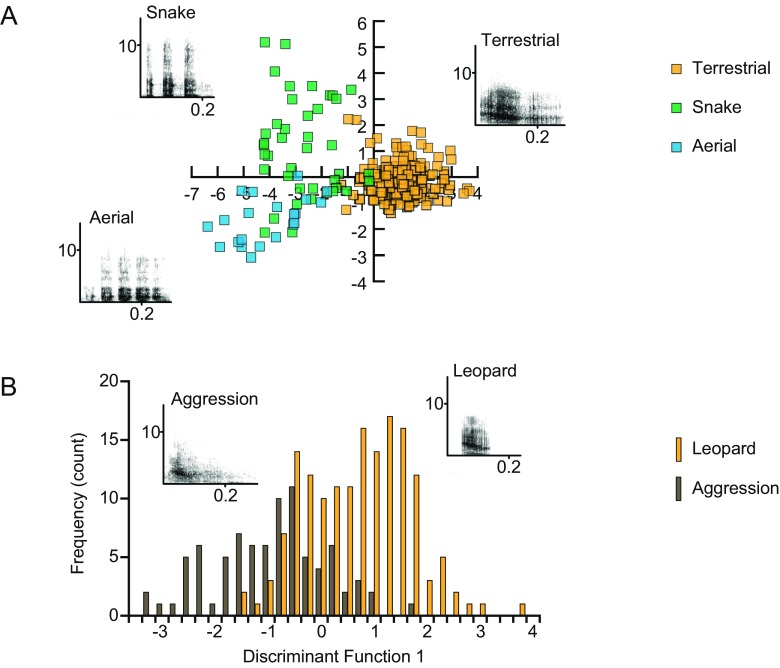
Structure and discriminability of male vervet vocalizations given in alarm and aggressive contexts. (**a**) Scatter plot of the discriminant scores with corresponding spectrograms of East African male alarm calls given in response to leopards, eagles, and snakes. (**b**) Frequency distribution of the discriminant scores with corresponding spectrograms of South African male vocalizations given in response to leopards and during within- and between-group aggression. *DF* discriminant function. Modified from Price et al., 2015

The finding that animals utter acoustically similar calls in different contexts, such as predator and aggressive contexts, is compatible with the idea that the calls reflect similar affective or motivational states (Price et al., 2015). One might object that it is unlikely that such different events as a between-group encounter and the detection of a snake would evoke similar affective or motivational states; on the other hand, if these events evoke dissimilar affective or motivational states, why would the animals give similar-sounding calls? Without independent evidence, it is impossible to settle this question. The idea that vocalizations are expressions of emotions is not only deeply entrenched in evolutionary theory (Darwin, 1872), but is also supported by considerations about the evolution of signals more generally, which posits that receivers attend to signals because they predict behavioural tendencies in the signaller, such as imminent attack (Maynard Smith & Harper, 2003). Alarm calls are a special case in this regard, because additional selection pressures, such as the benefits of advertising to the predator that it has been detected, may play a role (Zuberbühler, Jenny, & Bshary, 1999). Matters are further complicated by the fact that the categorization of events in the environment is subject to learning (Seyfarth & Cheney, 1980), making it difficult to relate specific events with specific motivational states. Furthermore, multiple factors, such as the presence of others as well as prior events, contribute to the likelihood and intensity of calling (Zuberbühler, 2000, 2008).

Ultimately, we would like to understand how the perception of the environment is linked to the utterance of calls. A study in which squirrel monkeys, *Saimiri sciureus*, could either seek or avoid electric stimulation of specific brain areas revealed that these two situations predictably elicited different call types, namely shriek cackles, shrieks and alarm peeps in aversive situations, and twitters, groans and chucks in positive contexts (Jürgens, 1979), supporting the view that calls are tied to different affective or motivational states. Future studies are needed to describe the neural pathways that couple the cognitive appraisal of a situation with specific motivational states, and ultimately the production of specific calls (Ackermann, Hage, & Ziegler, 2014).

Irrespective of whether or not primate calls are tied to specific motivational states, it seems that these utterances have little in common with linguistic reference in humans. My colleague Brandon Wheeler and I therefore felt there was a need to reconsider whether ‘functionally referential signalling’ is in any way special, or particularly informative for understanding language evolution. We argued that all evolved signals must have a certain degree of specificity (‘stand for something’), otherwise they would not be predictive of events or states and receivers would ignore them (Wheeler & Fischer, 2012). In this sense, signals classified as functionally referential are not qualitatively different from any other signals. In conclusion, the identification of (more or less) context-specific signals that elicit specific responses does not offer substantial insights into the evolution of semantic reference in human speech production. We therefore suggested abandoning the concept altogether (Wheeler & Fischer, 2012). Nevertheless, why some calls are more specific than others, and why some species have more differentiated call repertoires than others, remain intriguing issues. Comparative analyses that assess the influence of phylogenetic descent, habitat and predation pressure, as well as novel methods to describe the differentiation of repertoires will be needed to identify the factors that shape primate vocal repertoires (Fischer, Wadewitz, & Hammerschmidt, 2016). Intriguingly, for recipients, the processing of more ambiguous signals may effectively be more challenging than the processing of highly stereotyped and distinct signals (Wheeler & Fischer, 2012).

In light of the available evidence, I would maintain that we can now pretty much rule out the idea that nonhuman primates designate predators (or other events), or label them, at least if a label is understood as a conventionalized sign with an arbitrary relationship between the signifier and the signified. Instead, the structure of primate vocalizations is largely innate (see Hammerschmidt & Fischer, 2008, for a review).

Some of the controversies in this field are due to different interpretations of the same data. For instance, an analysis of the changes in the vocal structure of food grunts observed in one group of chimpanzees, which was merged with another group of chimpanzees, was taken as evidence *for* vocal learning (Watson et al., 2015b). Fischer, Wheeler, & Higham (2015) pointed out that there was substantial overlap in call structure to begin with, which would support the idea of an innate structure. Thus, the data could also be taken as evidence *against* (the necessity of) vocal learning. Possibly, the shift in frequency characteristics observed in some of the subjects reflected a certain degree of vocal accommodation (Fischer, 2002), which has been reported in a number primate species (Fischer et al., 2015; see also Watson et al., 2015a). Yet, it remains unclear why only some subjects of one group altered their calling characteristics but none of the other group members did so. Further studies with larger sample sizes would be needed to disentangle the contribution of social factors and auditory experience to assumed changes in vocal structure.

Studies on the variation in calling between different species of the same genus, or between subspecies, also suggest that the structure of nonhuman primate calls is highly conserved. The comparison of the acoustic structure of male barks of the genus *Chlorocebus* revealed only marginal differences between calls given by two different subspecies of vervet monkeys ranging in Eastern (*C. p. pygerythru*s) and Southern Africa (*C. p. hilgerti)*, respectively, and only marginally more pronounced differences between male calls of this species and the West African congener, *C. sabaeus* (Price, Ndiaye, & Fischer, 2014) (Fig. 3). Taken together, nonhuman primates do not have the capacity (nor necessity) to acquire their vocalizations; and hence they do not meet the minimal preconditions for developing a socially transmitted conventionalized communication system. Not being able to learn one’s sounds is not the only reason why animals lack language: neither songbirds, which learn their songs (Catchpole & Slater, 2008), nor apes that have been trained to use symbols or gestures, have acquired a proper language (Wallmann, 1992; Yang, 2013). Furthermore, there is also only scant evidence that primate signallers take into account the mental states of conspecifics, an important component of intentional communication (Scott-Philipps, 2015).

**Fig. 3:**
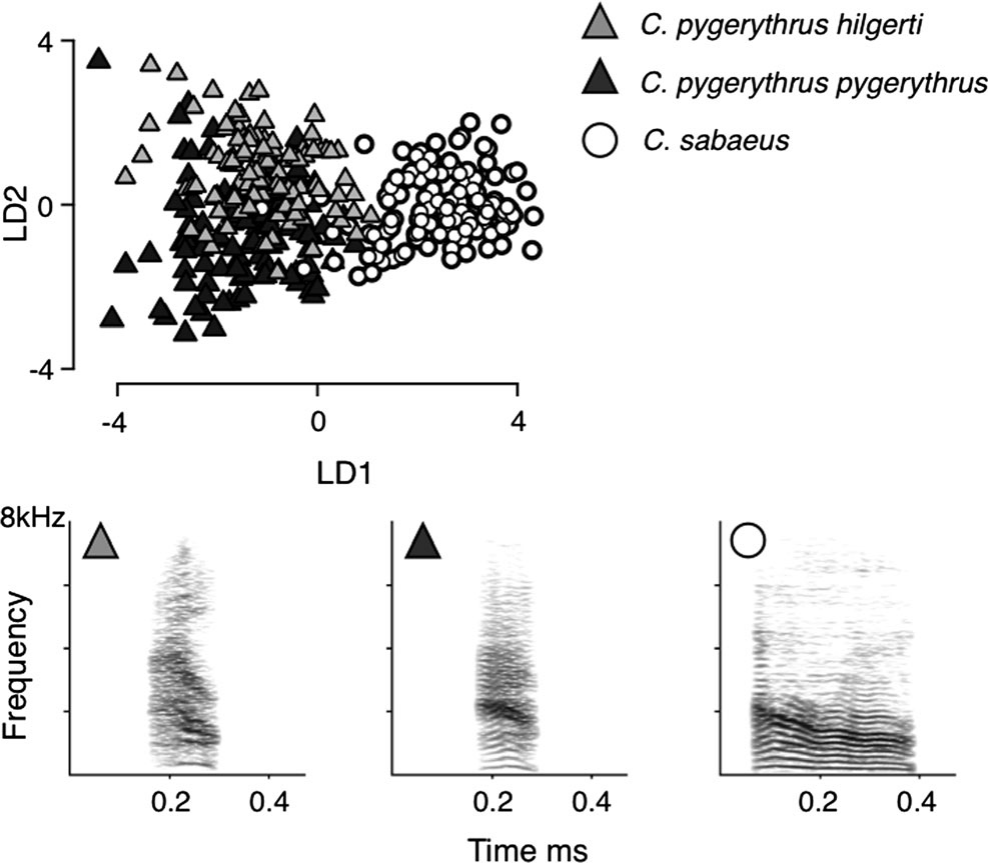
Scatterplot and spectrograms illustrating population differences in the acoustic structure of *C. sabaeus*, *C. p.hilgerti*, and *C. p. pygerythrus* barks. The scatterplot presents the distribution of the first and second LDA discriminant scores. Spectrograms illustrate a typical call exemplar for each call group, with typical calls defined as those that were most likely to be assigned by LDA to the correct caller/population. From Price et al., 2014

The limitations in terms of control over the acoustic structure do not apply to the usage of calls, nor to the receivers’ side. Nonhuman primates have some volitional control over the *usage* of their innate calls (Fischer et al., 2015; Watson et al., 2015b), but this does not entail control over call form or structure (Janik & Slater, 2000). Notably, rhesus monkeys, *Macaca mulatta*, can be trained to give one of two different types of food calls on command (Hage, Gavrilov, & Nieder, 2013), and volitional control over the onset of vocalizations is known from many primate studies (Larson, Sutton, Taylor, & Lindeman, 1973; Myers, Horel, & Pennypacker, 1965; Randolph & Brooks, 1967; Sutton, Larson, Taylor, & Lindeman, 1973). There is also good evidence that infant monkeys need to learn what certain signals stand for (see Fischer, 2002, for a review). Indeed, the cognitive processing of sounds by monkeys is quite sophisticated. Barbary macaques, *Macaca sylvanus*, categorize continuous variation in their alarm calls into different categories (Fischer, 1998); female putty-nosed monkeys, *Cercopithecus nictitans*, integrate information retrieved from conspecific calling sequences to predict the appearance of different predators or group movement (Arnold & Zuberbühler, 2008); and West African Green monkeys as well as putty-nosed monkeys factor in context when they respond to the playback of alarm calls (Arnold & Zuberbühler, 2013; Price & Fischer, 2014).

Notably, from the receiver’s point of view, it is entirely unimportant whether calls given in different contexts vary because they are tied to different emotional states, or whether they are acquired conventional signs (see Wheeler and Fischer 2012). What is important to the receiver is the degree to which calls can be used to make inferences about events in the environment. In sum, the continuity between nonhuman primate communication and speech is at the side of the processing and interpretation of the signals, while a deep trench separates humans from other primates at the level of the production of signals (Seyfarth & Cheney, 2003; and this issue). The dichotomy between perception and production in nonhuman primates is deeply puzzling, and studies of the gene networks orchestrating brain development may one day provide insights into the reorganization of the connectivity in the brain (Friederici, 2009; Rilling et al., 2008).

To be clear, I do not want to suggest that the search for evolutionary precursors of speech in nonhuman primate communication was in vain. We have learnt a great deal about the exquisite skills of receivers, who take in and weigh information from different sources, to make adaptive decisions (Fischer, 2013). We also know that nonhuman primates have a certain degree of volitional control over their vocalizations (see above). Thus, neither the processing of signals nor the control over usage appear to be the limiting factors in language evolution. But we also know now how striking the fundamental differences between nonhuman primate vocal production and speech production are. In addition to exploring the genetic basis for vocal learning (or the inability thereof; Fischer & Hammerschmidt, 2011; Fisher & Scharff, 2009), I feel the future of primate communication studies lies in some of the very basic evolutionary questions: What is the function and survival value of different primate signals? What is the role of sexual selection in primate vocal communication? And how do nonhuman primates get away with such limited repertoires, although they have such rich representations of the world?
